# Novel CoQ10 Antidiabetic Mechanisms Underlie Its Positive Effect: Modulation of Insulin and Adiponectine Receptors, Tyrosine Kinase, PI3K, Glucose Transporters, sRAGE and Visfatin in Insulin Resistant/Diabetic Rats

**DOI:** 10.1371/journal.pone.0089169

**Published:** 2014-02-20

**Authors:** Mohamed M. Amin, Gihan F. Asaad, Rania M. Abdel Salam, Hanan S. El-Abhar, Mahmoud S. Arbid

**Affiliations:** 1 Department of Pharmacology & Toxicology, Faculty of Pharmacy, Cairo University, Cairo, Egypt; 2 Department of Pharmacology, Medical Division, National Research Center, Cairo, Egypt; Consiglio Nazionale delle Ricerche, Italy

## Abstract

As a nutritional supplement, coenzyme Q10 (CoQ10) was tested previously in several models of diabetes and/or insulin resistance (IR); however, its exact mechanisms have not been profoundly explicated. Hence, the objective of this work is to verify some of the possible mechanisms that underlie its therapeutic efficacy. Moreover, the study aimed to assess the potential modulatory effect of CoQ10 on the antidiabetic action of glimebiride. An insulin resistance/type 2 diabetic model was adopted, in which rats were fed high fat/high fructose diet (HFFD) for 6 weeks followed by a single sub-diabetogenic dose of streptozotocin (35 mg/kg, i.p.). At the end of the 7^th^ week animals were treated with CoQ10 (20 mg/kg, p.o) and/or glimebiride (0.5 mg/kg, p.o) for 2 weeks. CoQ10 alone opposed the HFFD effect and increased the hepatic/muscular content/activity of tyrosine kinase (TK), phosphatidylinositol kinase (PI3K), and adiponectin receptors. Conversely, it decreased the content/activity of insulin receptor isoforms, myeloperoxidase and glucose transporters (GLUT4; 2). Besides, it lowered significantly the serum levels of glucose, insulin, fructosamine and HOMA index, improved the serum lipid panel and elevated the levels of glutathione, sRAGE and adiponectin. On the other hand, CoQ10 lowered the serum levels of malondialdehyde, visfatin, ALT and AST. Surprisingly, CoQ10 effect surpassed that of glimepiride in almost all the assessed parameters, except for glucose, fructosamine, TK, PI3K, and GLUT4. Combining CoQ10 with glimepiride enhanced the effect of the latter on the aforementioned parameters. Conclusion: These results provided a new insight into the possible mechanisms by which CoQ10 improves insulin sensitivity and adjusts type 2 diabetic disorder. These mechanisms involve modulation of insulin and adiponectin receptors, as well as TK, PI3K, glucose transporters, besides improving lipid profile, redox system, sRAGE, and adipocytokines. The study also points to the potential positive effect of CoQ10 as an adds- on to conventional antidiabetic therapies.

## Introduction

Prevalence of insulin resistant (IR)/type 2 diabetes nowadays depends on the slothful life style on one side and on the high intake of “Westernized” diet, which deemed to be the main environmental trigger [1], [2], on the other side.

Pathogenesis of this metabolic disorder emerges from a complicated interplay between several factors, including mitochondrial dysfunction that lies somewhere along a continuum from genetic to environmental abnormalities. Several studies have pointed to the paramount importance of the mitochondrial dysfunction in different metabolic disorders including IR/type 2 diabetes [3], [4]. Mitochondrial dysfunction was found to be an underlying mechanism and a complication of diabetes, with a major role played by oxidative stress [5]. Hence, treatment or supplemental strategies that contemplate on improving mitochondrial function and constraining oxidative stress might present important prospect.

Among the mitochondria-linked supplements is the Co-enzyme Q10 (CoQ10), a fat-soluble, vitamin like quinone commonly known as ubiquinone. It exists in the body in 2 forms; the oxidized form that acts as an electron carrier during the mitochondrial respiration and the reduced form that serves as an endogenous antioxidant [6]. Although CoQ10 is present in most tissues, yet the heart, liver, kidneys, and pancreas possess the highest concentrations [6].

CoQ10 is one of several nutritional components that have been recorded to be inadequate in diabetic states [6]–[9]. The CoQ10 deficiency is possibly due to the impaired mitochondrial substrate metabolism and/or increased oxidative stress [10] that may be linked with the impairment of β-cells and the development of IR [11].

To this end, CoQ10 appears to be an interesting component that merits supplementation in several diabetes/insulin resistant studies in order to assess its ability to counteract insulin-resistance associated metabolic disorders, and/or diabetes-related complications; studies that were carried either experimentally [6], [8], [12], [13] or clinically [7], [14]–[16].

Albeit several studies show controversial findings regarding the antidiabetic effect of CoQ10, yet those that support its positive effect did not explain the potential mechanisms, which was the goal of this study. Therefore, we have tested the influence of CoQ10 supplementation on metabolic alterations induced by a combination of high fat-high fructose diet (HFFD) and a single subdiabetogenic dose of streptozotocin (STZ) to offer overt diabetic rats. To verify the possible mechanisms that may underlie the antidiabetic/insulin sensitizing effect of CoQ10, the study shed light on the possible involvement of insulin receptors, tyrosine kinase (TK) and phosphatidylinositol 3 kinase (PI3K), as main members of the insulin downstream cascade. Besides, the current work evaluated the potential effect on adiponectin receptors, glucose transporters and myeloperoxidase, in the liver and soleus skeletal muscle. In addition, the effect of CoQ10 on the serum level of endogenous soluble receptor of advanced glycated end products (sRAGE) and 2 adipocytokines, *viz*., adiponectin and visfatin was studied. Glimepiride was used as the standard antidiabetic drug to compare the CoQ10 effect and to assess its potential importance when combined with the sulfonylurea derivative.

## Materials and Methods

### 1. Drugs and Chemicals

CoQ10 and STZ were purchased from Sigma-Aldrich Co. (St Louis, MO, USA) and glimepiride (Amaryl) from Sanofi-Aventis Co. (Zeitoun, Cairo, Egypt), while long-acting insulin (Monotard) was obtained from Novo Nordisk Co. (Copenhagen, Denmark). The diet ingredients, such as cholesterol, was procured from Panreac Quimica (Barcelona, Spain), fructose was obtained from El-Nasr Chemical Co. (Abou Zaabal, Cairo, Egypt), while lard was brought from commercial sources and other chemicals used were of analytical grades. Both glimepiride and CoQ10 was administered orally, with the first suspended in distilled water, and CoQ10 was dissolved in 1%Tween 80.

### 2. Animals

Adult male Wistar albino rats weighing 80–90 g were purchased from the National Research Center Laboratory (Cairo, Egypt) and were housed in standard polypropylene cages and kept under constant environmental conditions and equal light-dark cycles. Rats were acclimatized for 1 week and were fed commercially available rat normal pellet diet and water *ad libitum*, prior to the dietary manipulation.

#### 2.1 Ethics Statement

This study was carried out in accordance with the recommendations in the Guide for the Care and Use of Laboratory Animals of the National Institutes of Health. The protocol was approved by the Committee of the Faculty of Pharmacy, on the Ethics of Animal Experiments of the Cairo University (Permit Number: PT 290). All surgery was performed under deep sodium pentobarbital anesthesia and all efforts were made to minimize suffering.

### 3. Development of Insulin Resistant/Type 2 Diabetic Rats

The current model was adopted according to a previous study by Schaalan et al. [2]. Briefly, 90 rats were divided into two main groups, where in the first one animals were fed normal fat diet ([NFD], n = 10) to serve as the normal control group. In the second group (n = 80) animals received an in-house-prepared high-fat diet, combined with fructose in drinking water (20%) for a period of 6 weeks (HFFD). During the 6^th^ week the 2^nd^ group was injected a daily single dose of long-acting human insulin (Monotard) (0.5 IU/kg, i.p) to augment a state of IR. On the 7^th^ week a freshly prepared single sub-diabetogenic dose of STZ (35 mg/kg in citrate buffer, pH 4.5) was injected once after an overnight fasting to produce frank hyperglycemia. During the development of this model a regular estimation of the body weight (BW), food, fructose/water and caloric intake, as well as the levels of fasting serum glucose, triglycerides (TGs), total cholesterol (TC) and insulin were carried out. At the end of the 7^th^ week, animals showing blood glucose level between 200–350 mg/dl, hyperinsulinemia and hyperlipidemia were considered as insulin resistant/type 2 diabetic rats and were included in the study.

### 4. Oral Glucose Tolerance Test (OGTT)

At the end of the 6^th^ week and before the injection of STZ, the OGTT (2 g glucose/kg) was performed on 6 rats from each group after an overnight fasting. Blood droplets were withdrawn from the tip of the tail vein at 0 time and every 30 min along 2 hours, to assess the blood glucose level using test strips for blood glucose testing device (Lifescan, USA). Insulin resistance was reflected by the OGTT and was further confirmed by the fasting hyperinsulinemia and the HOMA-I value.

### 5. Experimental Design

Animals that were confirmed to be obese, insulin-resistant, and diabetic were randomly divided into four groups (n = 10–12 rats). The 1^st^ group served as the obese insulin-resistant/type 2 diabetic control, while the 2^nd^ and 3^rd^ groups received either glimepiride (0.5 mg/kg, p.o) [2] or CoQ10 (20 mg/kg, p.o) [17], and their combination was administered to the 4^th^ group. The NFD animals were set as the last 5^th^ group (n = 10) and received the vehicle to function as the negative control group. All treatments sustained for 2 weeks and the last dose of any treatment was given 24 h before the animals were euthanized. Rats were fasted 18 h before the time of death to minimize the feeding-related variations in the lipid and glucose patterns.

### 6. Collection of Serum Samples for Analysis

At the time of carnage, animals were weighed and blood was collected from the tail vein under brief ether anesthesia and was centrifuged (700×g, 4°C, 20 min) to separate sera. Sera were used to determine glucose, liver enzymes (AST and ALT) and malondialdehyde (MDA) as a measure for lipid peroxidation, using available colorimetric reagent kits [Randox (Antrim, U.K), Quimica Clinica Aplicada (Amposta, Spain) and Biovision (California, USA), respectively]. ELISA technique was carried out using the corresponding ELISA kit for the assessment of fructosamine (EIA ab, Wuhan, China), reduced glutathione (Cayman Chemical, Michigan, USA), adiponectin (Chemicon, MA, USA), the soluble receptor of advanced glycated end product (sRAGE) and visfatin (Ray Biotech, Georgia USA). Serum lipid profile, *viz.,* triglycerides (TGs), total cholesterol (TC) and free fatty acids (FFAs) were estimated using Synchron CX5 auto-analyzer (Beckman Instruments INC, Brea, USA). Finally, insulin level was measured using rat RIA kit (Sceti Medical Labo K.K, Tokyo, Japan), and Homeostasis Model Assessment-index (HOMA-index) was calculated according to the following equation [Fasting glucose (mg/dl)/18×Fasting insulin (µIU/ml)]/22.5 [18].

### 7. Tissue Extracts

Following blood collection animals were killed by a deep sodium pentobarbital anesthesia and both the liver and soleus skeletal muscle were dissected out, washed, dried, weighed, homogenized in PBS (10%) and kept in aliquots for the estimation of the following biomarkers.

#### 7.1. Assessment of lipid profile

One part of the aliquot was centrifuged at 1500×g at 4°C for 15 minutes and the supernatant was collected for the assessment of TGs, TC and FFAs using Synchron CX5 auto-analyzer (Beckman Instruments INC, Brea, USA).

#### 7.2. Assessment of adiponectin receptors, glucose transporters, and myeloperoxidase

Two other parts of the aliquots were exposed to 2 repeated freeze-thaw cycle to further break the cell membranes, then centrifuged at 5000×g for 5 minutes and stored at −20°C. These aliquots were used for the estimation of adiponectin receptor 1 and 2 (Adipo R1 & R2) [19], glucose transporter 2 and 4 (GLUT 2 & 4) and myeloperoxidase (MPO) activity, using the corresponding ELISA rat kit (Uscn, Wuhan, China; EIAab, Wuhan, China and Hycult biotech, Uden, Netherlands, respectively).

#### 7. 3. Assessment of the activities of tyrosine kinase and PI3K

These two parameters were assayed in one aliquot that was processed as mentioned under 7.2. The activity of both tyrosine kinase (TK) and PI3K was determined by the proper ELISA rat kit (Chemicon, Billerica, USA and Millipore, Massachusetts, USA, respectively).

#### 7.4. Protein Assay

The protein content in the liver and muscle tissue homogenates was assayed using the method described by Bradford [20] and the bovine serum albumin was used as a standard.

#### 7. 5. Assessment of High Affinity and Low Affinity Insulin Receptors (HAIR & LAIR)

HAIR and LAIR were measured using RIA method, where 2.5 ml aliquot of the sample was incubated in polystyrene tube (12×75 mm) with 3.37×10^−11^ of ^125^ I - labeled insulin in 200 µl of HEPES buffer (25 mM, pH 7.8). The latter contains 0.1% Triton X-100, 150 mM sodium chloride, 1% bovine serum albumin (BSA), 100 U/ml bacitracin and varying concentrations of unlabeled insulin for 16 hours at 4°C. At the end of incubation, 100 µl of 0.3% gamma globulin (w/v) and 300 µl of 25% polyethylene glycol (w/v, molecular weight 8000) were added to each tube and all tubes were left for another 20 minutes on ice bath. Tubes were then centrifuged at 10,000×g for 10 minutes at 4°C to separate the bound hormone- receptor complex from the free hormone. After centrifugation, careful, decantation was carried out and the pellets were washed with 300 µl of 12.5% of the same polyethylene glycol and centrifuged again at 10,000×g for 10 minutes at 4°C. The resulting pellets were counted for two minutes in a gamma counter to get the total binding of each tube. Specific binding was determined by the difference between total binding and non-specific binding and the total count was determined for each assay (it is the count of any tube before centrifugation). A control tube was run with each assay, where it was passed through the same steps like all other tubes except that the ^125^ I- labeled insulin was added just before centrifugation. All counts were corrected for the control tube count before any calculation. The data were analyzed for insulin receptors concentration and dissociation constant (KD) [21].

### 8. Statistical Analysis

Values are expressed as mean ± S.E. of 8–10 animals and the differences between groups were tested for significance using analysis of variance (ANOVA), followed by Tukey-Kramer post-hoc test determined by SPSS software program, version 21. Correlation coefficient (r) was carried using linear regression analysis. The level of statistical significance was taken at *P*<0.05. The graphs were drawn using a prism computer program (GraphPad software Inc. V5, San Diego, CA, USA).

## Results

**Figure 1:**
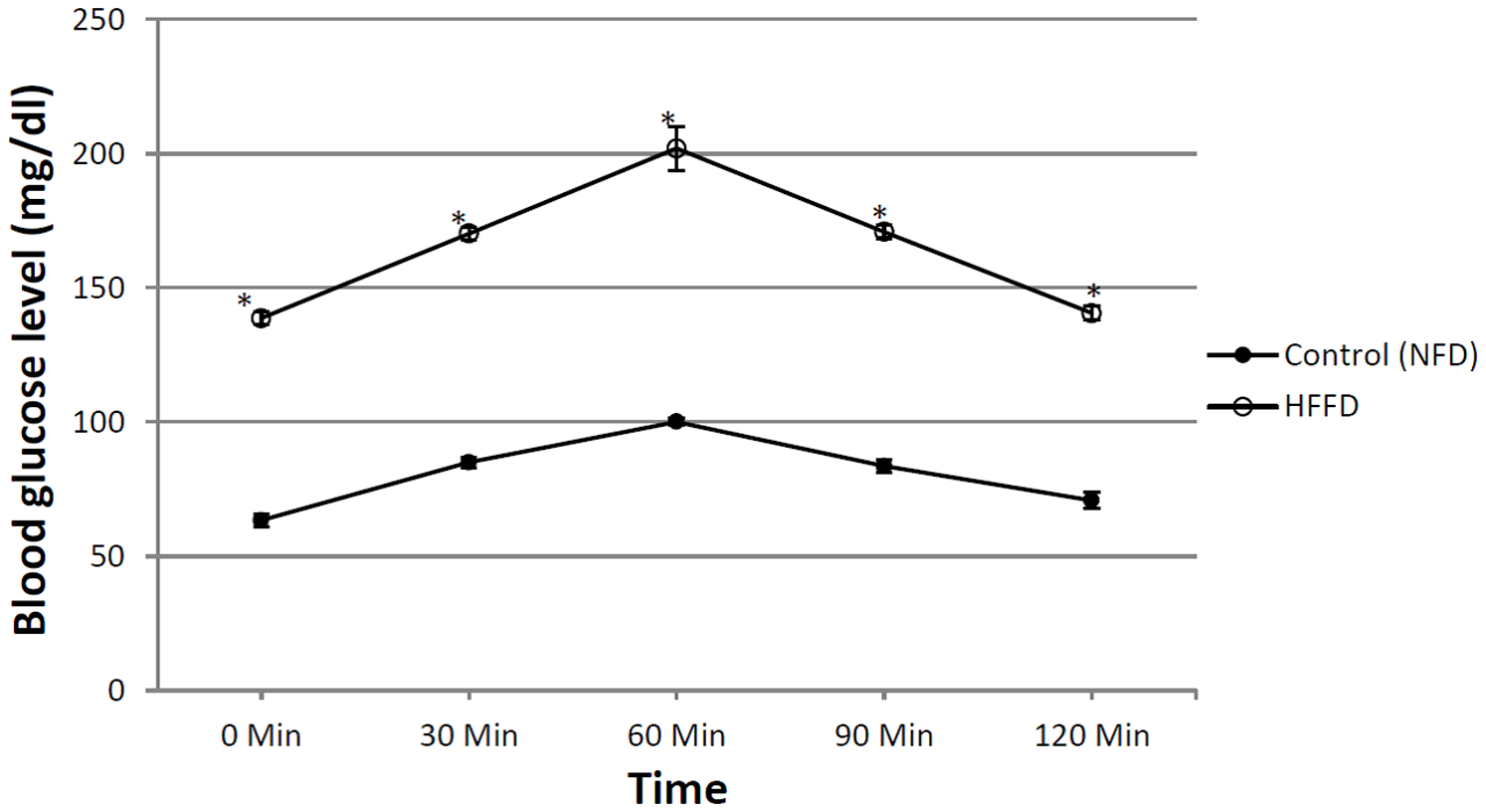
The glucose tolerance curve depicts the changes in glucose (2 g/kg, p.o) response in serum of normal control group (NFD) and non-treated insulin-resistant rats (HFFD), after 6 weeks of food manipulation at 0, 30, 60, 90, and 120 min. Values are means ± S.E of 6 animals; as compared to the normal control group (*) (one-way ANOVA followed by Tukey-Kramer post hoc test), *P*<0.05.

**Figure 2:**
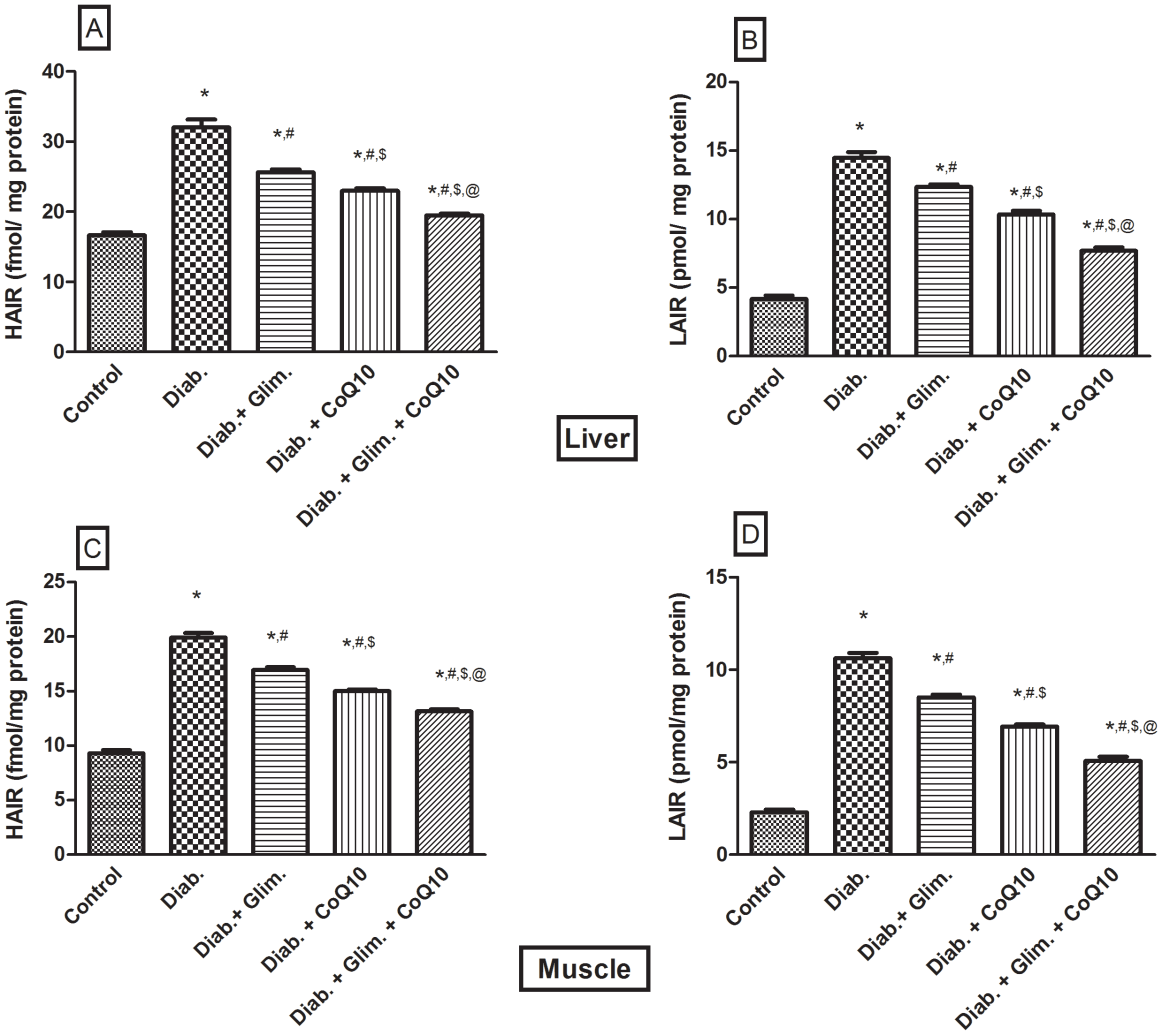
Effect of diabetes and 2 weeks oral administration of CoQ10 (20 mg/kg) and/or glimepiride (0.5 mg/kg) on the hepatic (A, B) and muscular (C, D) insulin receptor isoforms (high affinity, [HAIR, fmol/mg protein] and low affinity [LAIR, pmol/mg protein] insulin receptor). Values are means of 8–10 animals ± S.E.M. As compared with normal control (*), diabetic control (^#^), glimepiride treated (^$^) and CoQ10 treated (@) groups (one-way ANOVA followed by Tukey-Kramer post hoc test), *P*<0.05.

**Figure 3:**
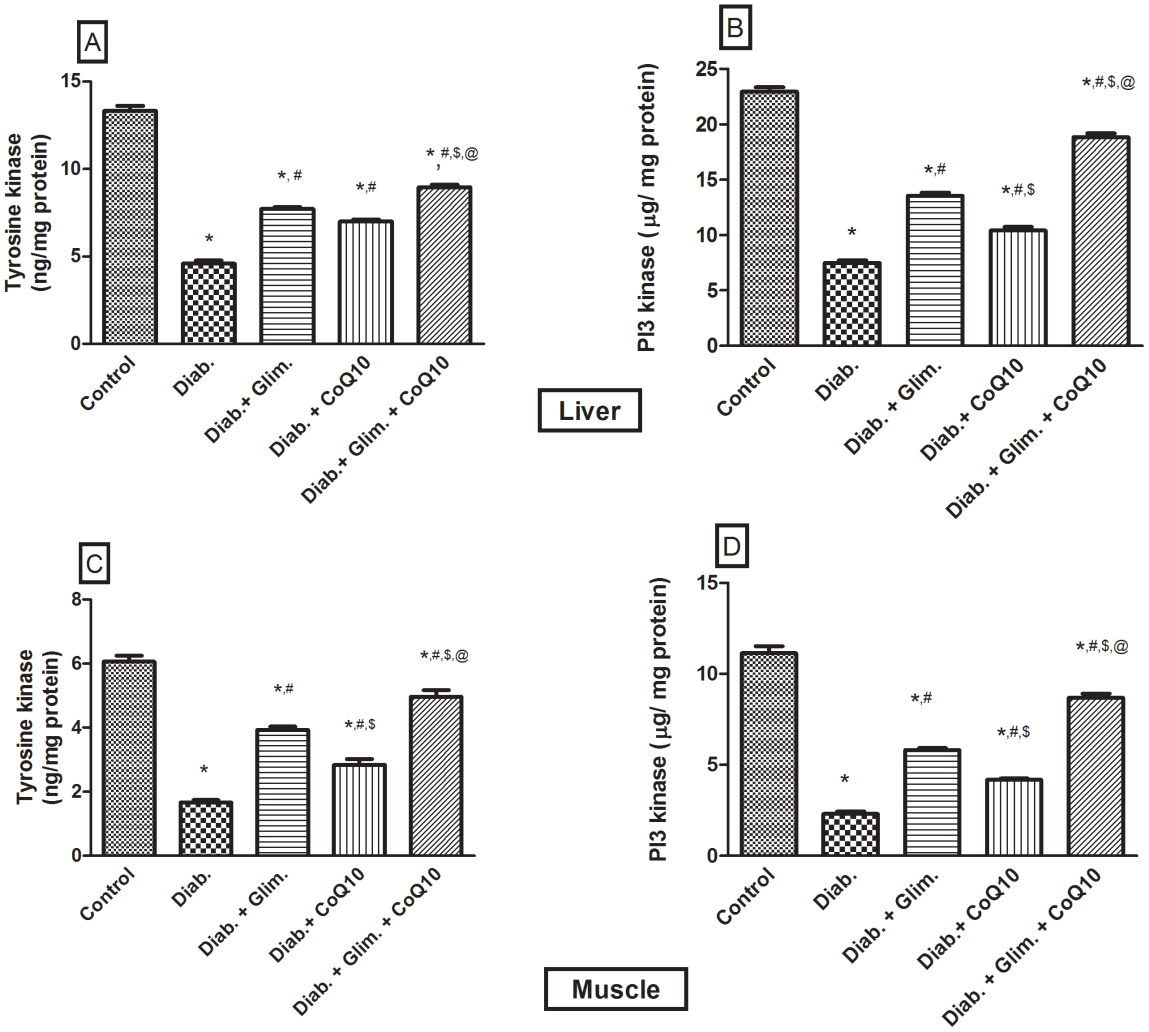
Effect of diabetes and 2 weeks oral administration of CoQ10 (20 mg/kg) and/or glimepiride (0.5 mg/kg) on the hepatic (A, B) and muscular (C, D) activity of tyrosine kinase (ng/mg protein) and PI3K (µg/mg protein). Values are means of 8–10 animals ± S.E.M. As compared with normal control (*), diabetic control (^#^), glimepiride treated (^$^) and CoQ10 treated (^@^) groups (one-way ANOVA followed by Tukey-Kramer post hoc test), *P*<0.05.

**Figure 4:**
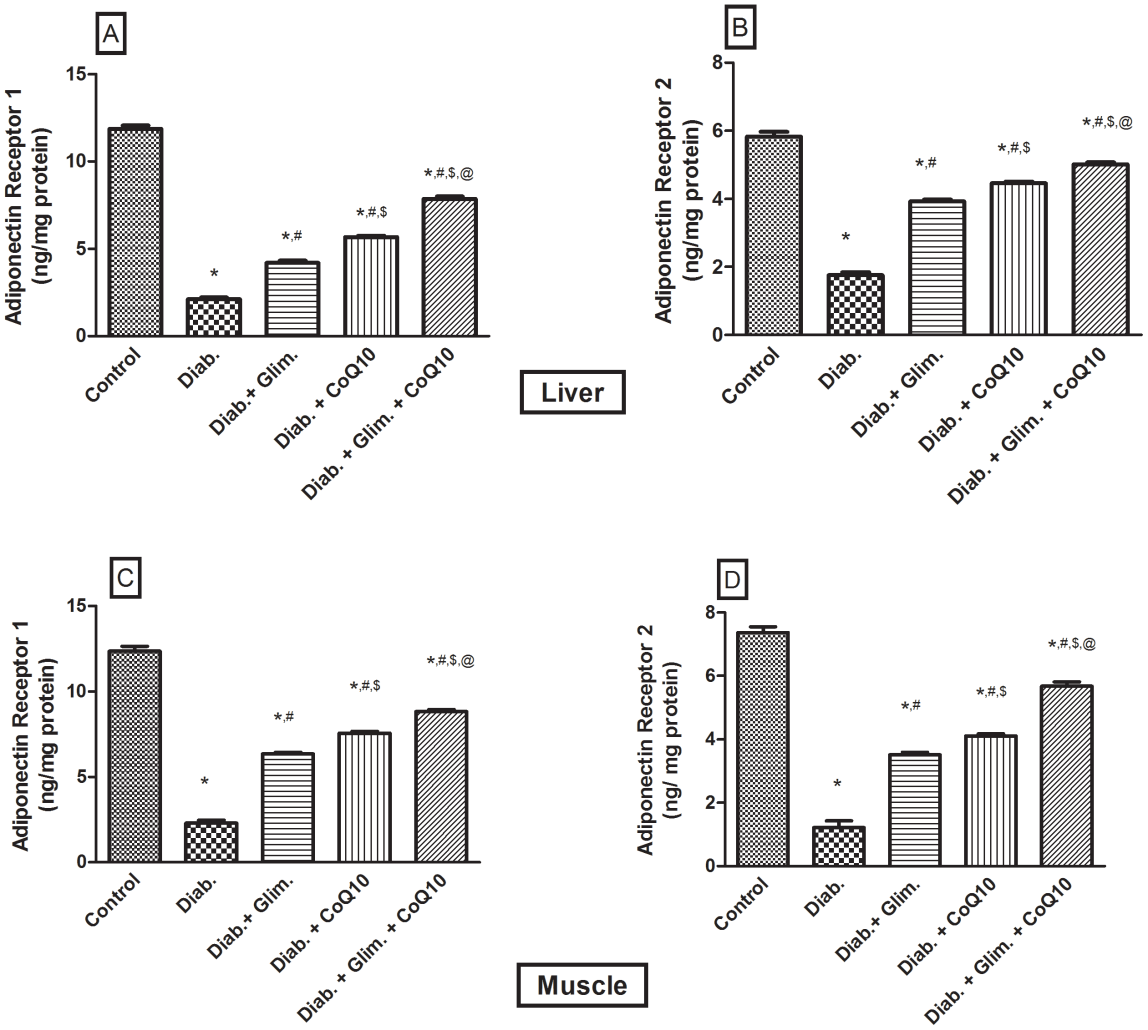
Effect of diabetes and 2 weeks oral administration of CoQ10 (20 mg/kg) and/or glimepiride (0.5 mg/kg) on hepatic (A, B) and muscular (C, D) adiponectin receptors (Adipo-R1, Adipo-R2 [ng/mg protein]). Values are means of 8–10 animals ± S.E.M. As compared with normal control (*), diabetic control (^#^), glimepiride treated (^$^) and CoQ10 treated (^@^) groups (one-way ANOVA followed by Tukey-Kramer post hoc test), *P*<0.05.

**Figure 5:**
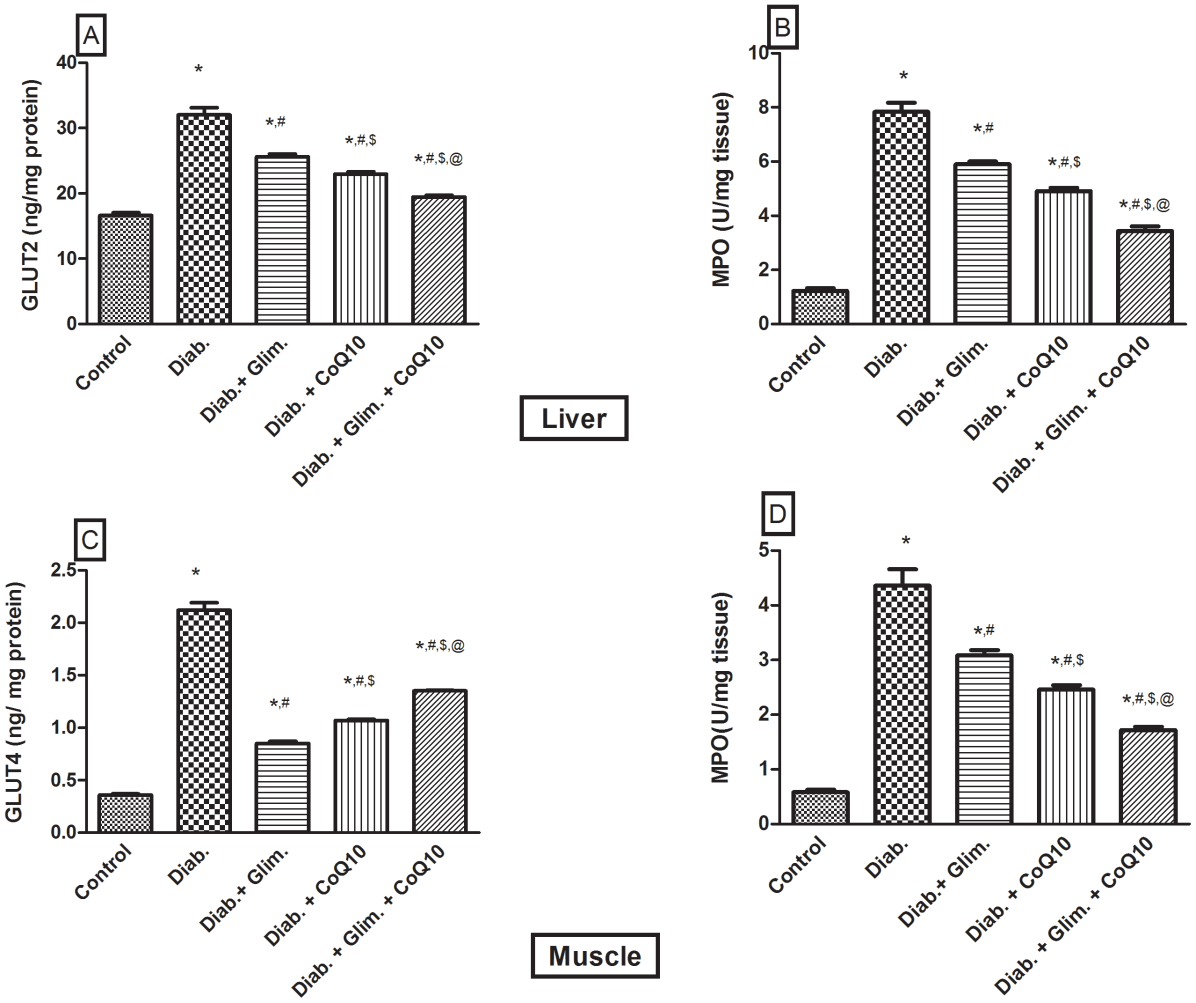
Effect of diabetes and 2 weeks oral administration of CoQ10 (20 mg/kg) and/or glimepiride (0.5 mg/kg) on the hepatic (A) and muscular (C) glucose transporters [ng/mg protein], and (B, D) myeloperoxidase activity [MPO, U/mg]. Values are means of 8–10 animals ± S.E.M. As compared with normal control (*), diabetic control (^#^), glimepiride treated (^$^) and CoQ10 treated (^@^) groups (one-way ANOVA followed by Tukey-Kramer post hoc test), *P*<0.05.

Table 1 showed that the HFFD resulted in a significant increase in the BW compared to the 1^st^ week; however, the difference in the BW from the 5^th^ week to the 7^th^ one was not significant. The food intake was significantly increased in the 6^th^ and 7^th^ weeks, but not the 5^th^ one. Moreover, the intake of the 20% fructose in water was increased in the 5^th^ and 6^th^ weeks as compared with the 4^th^ and 7^th^ weeks. The food and fructose/water intake was reflected on the total Kcal/day, where in the 7^th^ week, during which animals drank water only, was significantly the lowest as compared to the 1^st^, 5^th^ and 6^th^ weeks. Furthermore, the caloric intake in the 5^th^ and 6^th^ weeks was significantly higher than that in the 1^st^ one. It should be noticed that during the 7^th^ week the animals received NFD and water instead of the HFFD consumed in the previous 6 weeks. In figure 1 the OGTT showed that the HFFD effect presented the same pattern offered by the NFD along the 2 hours period, but with about 2 folds elevation indicating a state of glucose intolerance/insulin resistance. In relation to the normal control group, the untreated diabetic group showed a discernible increase in serum level of glucose, fructosamine, insulin, and HOMA-I value (Table 2). The model also induced conspicuous increment in the serum, liver and muscle levels of TGs (147, 433 & 313%, respectively), TC (90, 365 & 273%, respectively), and FFAs (3.4, 4.2 & 3.4 folds, respectively) and doubled that of ALT and AST as presented in table 3. HFFD/STZ also increased MDA by1.7 folds, as well as the adipocytokine visfatin, but it conversely demonstrated a sharp decline in the serum level of adiponectin (88%), glutathione (83%) and sRAGE (32.7%) (Table 4). On the molecular level, the model boosted the insulin receptor isoforms in the liver and muscle (Figure 2 A–D), but inhibited noticeably those of tyrosine kinase and PI3K in both tissues (Figure 3 A–D), as well as adiponectin receptors (Figure 4 A–D). The model also, as depicted in figure 5, increased the glucose transporters in both the liver (GLUT2, panel A) and muscle (GLUT4, panel C), as well as the MPO activity in both tissues (Panels B and D).

**Table 1:** The assessment of food and fructose/water intake, and body weight of the rats before and at the end of the HFFD.

	Body weight(g)	Food intake[HFD] (g)	Food intake[NFD] (g)	Fructose (20%)/water intake (ml)	Water intake(ml)	TotalKcal/day
**1^st^ Week**	81.6±1.12	29.70±1.41	–	43.6±1.51	–	183.57±8.37
**5^th^ Week**	290.5±3.58*	34.68±2.14	–	54.1±3.52*^#^	–	216.26±12.7^#^
**6^th^ Week**	297.3±3.6*	39.21±2.66*	–	58.5±2.41*^#^	–	242.9±15.54*^#^
**7^th^ Week**	290.2±3.24*		37.19±1.6*	^-^	38.9±2.03	117.14±5.04*

Values are presented as means **±** S.E.M. (n = 70–80). Animals were fed high fat diet [HFD] and were allowed free access to 20% fructose in water throughout the 6 weeks. Diet manipulation was stopped after the STZ injection on day 1 of the 7^th^ week. During this week animals received normal fat diet [NFD] and water. Caloric intake for NFD = food intake (g) × 3.15 kcal, caloric intake for HFD = food intake (g) × 5.3 kcal and caloric intake for 20% fructose = 0.6 kcal/ml. As compared with 1^st^ week (^*^), and 7^th^ week (^#^) (one-way ANOVA followed by Tukey-Kramer post hoc test), *P*<0.05.

**Table 2:** Effect of 2 weeks daily oral administration of CoQ10 (10 mg/kg) and/or glimepiride (0.5 mg/kg), on serum levels of glucose, fructosamine, insulin, and HOMA-index in HFFD/STZ-induced diabetic rats.

	Normal Control(NFD)	Diabetic Control(HFFD/STZ)	Diabetic+Glimepiride	Diabetic+CoQ10	Diabetic+Glimepiride/CoQ10
**Glucose (mg/dl)**	81±1.97	324.60±8.47*	213.22±5.36*^,#^	286.10±3.28*^,#,$^	210.12±5.32*^,#,@^
**Fructosamine (nmol/l)**	9.09±0.66	70.17±3.65*	22.97±0.75*^,#^	29.38±0.42*^,#,$^	33.62±0.45*^,#,$^
**Insulin (µIU/ml)**	1.61±0.14	44.98±2.50*	27.53±1.10*^,#,@^	19.46±0.57*^,#,$^	14.15±0.51*^,#,$,@^
**HOMA-index**	0.38±0.04	34.47±1.65*	14.45±0.55*^,#^	13.77±0.37*^,#^	7.35±0.37*^,#,$,@^

Values are means ± S.E of 8–10 animals. As compared with normal control (*), diabetic control (^#^), glimepiride treated (^$^) and CoQ10 treated (^@^) groups (one-way ANOVA followed by Tukey-Kramer post hoc test), *P*<0.05.

**Table 3:** Effect of 2 weeks daily oral administration of CoQ10 (10 mg/kg) and/or glimepiride (0.5 mg/kg), on serum, hepatic and muscular contents of triglycerides (TGs), total cholesterol (TC) and free fatty acids (FFAs), as well as serum ALT and AST in HFFD/STZ diabetic rats.

	Normal Control(NFD)	Diabetic Control(HFFD/STZ)	Diabetic+Glimepiride	Diabetic+CoQ10	Diabetic+Glimepiride/CoQ10
**Serum TGs (mg/dl)**	66.8±2.80	164.91±2.8*	142.28±2.18*^,#^	123.9±1.6*^,#,$^	109.57±1.56*^,#,$,@^
**Liver TGs (mg/g tissue)**	2.9±0.15	15.92±0.6*	11.68±0.25*^,#^	9.65±0.21*^,#,$^	7.27±0.58*^,#,$,@^
**Muscle TGs (mg/g tissue)**	5.5±0.34	22.75±0.9*	19.35±0.40*^,#^	16.32±0.36*^,#,$^	11.57±0.47*^,#,$,@^
**Serum TC (mg/dl)**	91.9±3.10	175.6±2.9*	151.84±2.48*^,#^	133.41±1.69*^,#,$^	121.06±0.23*^,#,$,@^
**Liver TC (mg/g tissue)**	3.5±0.19	16.37±0.6*	11.96±0.19*^,#^	9.71±0.20*,^#,$^	7.31±0.23*^,#,$,@^
**Muscle TC (mg/g tissue)**	6.4±0.34	23.82±0.8*	20.41±0.40*^,#^	17.42±0.36*^,#,$^	12.55±0.48*^,#,$,@^
**Serum FFA (mg/dl)**	14.5±1.43	50.05±3.7*	29.11±1.9*^,#^	36.81±1.4*^,#,$^	21.56±1.33*^,#,$,@^
**Liver FFA (mg/g tissue)**	4.0±0.27	16.88±0.6*	12.77±0.25*^,#^	10.76±0.21*^,#,$^	8.37±0.23*^,#,$,@^
**Muscle FFA (mg/g tissue)**	7.5±0.35	25.95±0.8*	21.68±0.46*^,#^	18.52±0.36*^,#,$^	13.63±0.48*^,#,$,@^
**AST (IU/l)**	35.9±0.43	86.23±0.7*	73.32±1.20*^,#^	69.77±0.98*^,#^	55.88±2.34*^,#,$,@^
**ALT (IU/l)**	20.9±0.42	53.37±0.4*	49.66±0.47*^,#^	38.36±0.49*^,#,$^	45.01±0.60*^,#,$,@^

Values are means ± S.E of 8–10 animals. As compared with normal control (^*^), diabetic control (^#^), glimepiride treated (^$^) and CoQ10 treated (^@^) groups (one-way ANOVA followed by Tukey-Kramer post hoc test), *P*<0.05.

**Table 4:** Effect of 2 weeks daily oral administration of CoQ10 (10 mg/kg) and/or glimepiride (0.5 mg/kg) on serum level of reduced glutathione (GSH), malondialdehyde (MDA), adiponectin, visfatin and soluble receptor of advanced glycated end product (sRAGE) in HFFD/STZ diabetic rats.

	Normal Control(NFD)	Diabetic Control(HFFD/STZ)	Diabetic+Glimepiride	Diabetic+CoQ10	Diabetic+Glimepiride/CoQ10
**GSH (µM)**	14.64±0.05	2.45±0.23*	8.80±0.13*^,#^	12.61±0.24*^,#^	12.69±0.55*^,#,$^
**MDA (nmol/ml)**	8.88±0.08	15.29±0.32*	11.99±0.14*^,#^	11.09±0.09*^,#,$^	9.98±0.34*^,#,$,@^
**Adiponectin (ng/ml)**	5.21±0.11	0.64±0.018*	2.47±0.19*^,#^	3.84±0.11*^,#,$^	5.04±0.25^#,$,@^
**Visfatin (ng/ml)**	15.28±2.05	201.55±4.73*	156.60±2.21*^,#^	128.57±1.85*^,#,$^	96.26±.96*^,#,$,@^
**sRAGE (ng/ml)**	1.77±0.08	1.19±0.03*	2.61±0.13*^,#^	3.44±0.03*^,#,$^	3.88±0.08*^,#,$,@^

Values are means ± S.E of 8–10 animals. As compared with normal control (*), diabetic control (^#^), glimepiride treated (^$^) and CoQ10 treated (^@^) groups (one-way ANOVA followed by Tukey-Kramer post hoc test), *P*<0.05.

As presented in table 2, treatment with CoQ10 alone for 2 weeks repressed the insulin level (56.7%), HOMA-I (60%) and fructosamine (58.1%), when compared to the diabetic group, with subtle, yet significant effect on the glucose level (11.8%). CoQ10 abated the model-mediated lipid profile elevation in serum, liver and muscle, as well as the two aminotransferases (Table 3); this effect entailed the pro-inflammatory cytokine, visfatin and the MDA level (Table 4). On the other hand, and as presented in the latter table, CoQ10 boosted the levels of adiponectin (6 folds), GSH (5 folds) and sRAGE (2.8 folds). Combining CoQ10 with glimepiride improved the previous parameters, an effect that was prominent in serum adiponectin (table 4), where it reached that of the NFD control group.

In the liver, the CoQ10 treated groups showed a 28% decrease in the levels of insulin-receptor isoforms (Figure 2 A, B), 25.4% in the GLUT2 by (Figure 5A), and 37.3% in the MPO (Figure 5B); the same pattern was observed in the skeletal muscle (Figures 2 C, D; 5 C, D). In figure 3, the four panels showed an elevation in the activity of both enzymes after the treatment with CoQ10, glimepiride and the combination regimen. The activities were increased in the same order and parallel the improvement in the glucose level. Additionally, CoQ10 caused about 60–70% increase in the adiponectin receptors in the liver (Figure 3 A, B) and muscle (Figure 3 C, D) compared to the non-treated diabetic group. As detected in tables 2–4 and figures 2–5, CoQ10 overrides the effects of glimepiride, in the redox parameters, lipid profile, inflammatory parameters and the molecular markers, except for the glucose, fructosamine, serum FFAs, TK, PI3K, and GLUT4 levels. When CoQ10 was added to the antidiabetic drug, a further improvement was observed in most of the biochemical markers, as well as in the molecular parameters (Tables 2–4; Figures 2–5).

Using the linear regression analysis, HOMA-index correlated negatively with the liver and muscle adiponectin receptors (Adipo-R1, Adipo-R2), as well as tyrosine kinase and PI3K. Conversely, a positive correlation was detected between the HOMA-I value and the glucose transporters [GLUT2/GLUT4] and insulin receptors [LAIR/HAIR] (P<0.001). On the other hand, adiponectin correlated negatively with GLUT2/GLUT4, and insulin receptors [HAIR and LAIR], while positively with its receptors (P<0.001), and both tyrosine kinase and PI3K, as depicted in tables 5 and 6.

**Table 5:** Correlation coefficient (r) between HOMA-index and serum adiponectin level with hepatic GLUT2, adiponectin receptors (Adipo- R1, R2), insulin receptor isoforms (HAIR, LAIR), tyrosine kinase (TK) and PI3K.

	GLUT2	Adipo-R1	Adipo-R2	HAIR	LAIR	TK	PI3K
**HOMA-I**	0.905 P<0.001	−0.882 P<0.001	−0.957 P<0.001	0.911 P<0.001	0.883 P<0.001	−0.886 P<0.001	−0.876 P<0.001
**Adiponectin**	−0.890 P<0.001	0.882 P<0.001	0.952 P<0.001	−0.939 P<0.001	−0.884 P<0.001	−0.804 P<0.001	0.847 P<0.001

Correlation was carried out in untreated and treated HFFD-STZ diabetic rats.

**Table 6:** Correlation coefficient (r) between HOMA-index and serum adiponectin level with muscular GLUT4, adiponectin receptors (Adipo- R1, R2), insulin receptor isoforms (HAIR, LAIR), tyrosine kinase (TK) and PI3K.

	GLUT4	Adipo-R1	Adipo-R2	HAIR	LAIR	TK	PI3K
**HOMA-I**	0.858 P<0.001	−0.947 P<0.001	−0.934 P<0.001	0.908 P<0.001	0.911 P<0.001	−0.890 P<0.001	−0.887 P<0.001
**Adiponectin**	−0.666 P<0.001	0.894 P<0.001	0.912 P<0.001	−0.900 P<0.001	−0.904 P<0.001	0.826 P<0.001	0.832 P<0.001

Correlation was carried out in untreated and treated HFFD-STZ diabetic rats.

## Discussion

Although CoQ10 has been extensively tested in insulin resistant/diabetic models and patients, yet the current study is the first to address new machineries that may underline its insulin sensitizing effect. CoQ10 herein revoked the HFFD/STZ effects on the content/activity of glucose/lipid panels, insulin receptors**,** tyrosine kinase, PI3K, adiponectin and its receptors, glucose transporters, visfatin, MPO and sRAGE. Moreover, the study showed that apart from being an insulin secretagogue, glimepiride can act *via* other mechanisms, where it played a significant role on the aforementioned parameters.

CoQ10 is known to be deficient in case of diabetes [6], [7] and in some individuals with reduced energy expenditure and skeletal muscle dysfunction [22]. Ample of evidence has shown that CoQ10 is proficient to counteract several metabolic disturbances associated with IR and diabetes [23]–[24], a fact that applies to the present findings. Mitochondrial respiratory capacity was reported to be reduced by the consumption of high fat diet, besides the aforementioned effects [25]; hence, the obliging effect of CoQ10 relies partly on its pivotal role in maintaining mitochondrial function [26] and on its ability to improve the lipid profile in serum, liver and muscle tissue as detected in the current work. Exposure of the liver to this type of diet not only elevates TGs, TC and favors FFAs esterification, but also accelerates lipogenesis with the accumulation of TGs that contribute, sequentially, to IR/glucose intolerance [27]. This phenomenon enhances the over-production of hepatic very low density lipoprotein, with the consequent increase in its chain connected lipoproteins [28], the *de-novo* lipogenesis under the influence of high carbohydrate feeding and lipolysis in adipose tissue leading to a high flux of FFAs [28], [29]. Moreover, elevated TC can abate the activation of hepatic cholesterol-rich lipoprotein (LDL-C) receptors and/or increases their glycation, resulting in its decreased catabolism [30].

The present model-induced dyslipidemia and precisely the elevated FFAs are responsible partly for the impairment of glucose uptake/utilization with the subsequent increase in hepatic glucose production [2], [31]. Furthermore, the increased blood glucose level plays a role in the IR induction through its flux into the hexosamine pathway [32], which expresses IR as an upshot of lipids and carbohydrates utilization imbalance. Therefore, lowering FFAs, TGs and TC is one vital arm in the CoQ10-mediated insulin sensitivity documented by the reduced HOMA-I and insulin level, as compared to the diabetic non-treated group. A similar effect was observed in the glimepiride treated rats and was more pronounced when combined with CoQ10.

Additionally, high fructose over nutrition not only induces the hexosamine pathway [33], but also reduces both insulin-stimulated autophosphorylation and insulin receptor substrate (IRS) phosphorylation in both liver and muscle [34], as documented herein by the inhibited tyrosine kinase activity and PI3K. These mechanisms alter the proper insulin function, a fact that was reflected here on the insulin receptors, which were increased in both organs, possibly as a corollary to the impaired insulin task. This finding was previously documented in liver [19] and skeletal muscles [35], [36] of prediabetic/diabetic models. Moreover, Sbraccia et al. [37] showed also that in case of IR the expression of LAIR correlates positively with hyperinsulinemia and negatively with insulin sensitivity. CoQ10 by elevating the activity of both tyrosine kinase and PI3K is responsible for improving the insulin cascade. These two enzymes are responsible for the insulin-stimulated autophosphorylation and its downstream flow, which was paralleled by the inhibition of the LAIR and HAIR content. This effect was even better when CoQ10 was combined with glimepiride. Regarding the effect of glimepiride on the insulin receptors we reported a decrease in their levels, possibly due to the improved insulin sensitivity, decreased FFAs, and/or the increased affinity and binding of insulin to its receptors as previously reported [38].

Revert to the hexosamine pathway glucose is diverted from the glycolytic pathway at the level of fructose-6-phosphate *via* the glutamine fructose-6-phosphate amidotransferase enzyme, with the consequent production of glucosamine-6-phosphate and other hexosamine products. As an inducer of IR, glucosamine inhibits the insulin-mediated glucose transport and glucose transporter 4 (GLUT4) translocation [39]. In the present study, however, the content of GLUT4 was increased in skeletal muscle, a finding that is debatable, where some studies showed a negative correlation [40], [41], while others [42], [43], including the present work, showed a positive correlation. We infer that GLUT4 elevation could be a compensatory reflex for serum hyperglycemia, yet failure of this reparation may be attributed to the impaired GLUT4 translocation to the cell membrane by the IR insult [39]. Previous study by Kruszynska et al. [44] assents with our results, where they stated that the elevated non esterified fatty acids, documented herein, induce IR and impair IRS-1 tyrosine phosphorylation and IRS-1-associated PI3K activation. Hence, the defected insulin signaling pathway can be responsible for the limited muscle glucose uptake and the impaired glucose transport. CoQ10, glimepiride and their combination have inhibited GLUT4 content in the skeletal muscles. This effect is linked to their ability to improve insulin signaling trail by increasing the activity of both tyrosine kinase and PI3K and the subsequent translocation of GLUT4 to cell membrane as evidenced by the decreased glucose level by CoQ10 and/or glimepiride. Previously, CoQ10 [45] and glimepiride [46] have been reported to activate the PI3K enzyme and its pathway in models other than diabetes.

Likewise, hepatic glucose transporter GLUT2 was also increased in the diabetic rats, a finding that coincides with previous studies [47], [48]. In contrast, in an earlier work with HFFD-induced IR without overt diabetes [19], hepatic GLUT2 leveled off, a finding that concur with that of Burcelin et al. [48], who reported that GLUT2 increases with hyperglycemia and decreases with hyperinsulinemia. Postic et al. [49] further clarified that when insulin and glucose are associated the stimulatory effect of glucose on GLUT2 gene expression predominates. CoQ10, as well as glimepiride and their combination brought the GLUT2 level down, *via* improving the hyperglycemia/hyperinsulinemia-associated metabolic disorders including dyslipidemia and the activation of PI3K. IRS-1 tyrosine phosphorylation, modulates on one hand the GLUT2 function to enhance the glucose uptake and on the other hand, it elevates the level of PI3K proven in this work. The downstream of the PI3K is the activation of Akt, which blocks gluconeogenesis and mediates glycogen synthesis [50].

Consequent to the hyperglycemia/IR, and according to the hyperglycemic extent, the formation of polyols and non enzymatic glycoxidation products of proteins and lipids occur, leading to the formation of heterogeneous products termed advanced glycation end-products (AGEs) [51] that are also associated with IR [52]. Although AGEs were not measured directly, yet their soluble receptor type (sRAGE), which reflects the activity of AGE/RAGE axis, decreased greatly in the current model and others [53], possibly as a consequence of eliminating the circulating AGEs burden. This receptor type was described previously as a decoy domain receptor that plays a role in blocking the AGE/RAGE interaction [54], [55]. The latter was reported to stimulate certain signaling cascade that leads to the induction of NAD(P)H oxidase with the consequential increase in reactive oxygen species (ROS) [55]. In a mutual scenario, Anderson et al. [56] stated also that AGEs may be generated by oxidative stress and inflammation, where MPO can trigger the production of RAGE ligands by generating different stresses, including NF-kB, which begets further inflammation and an endless cycle of AGE production. This fact was imitated herein by the marked activation of MPO, decreased GSH level and the 2 fold elevation of the lipid peroxide product and as documented previously [57], [58]. These results suggest that hyperglycemia may bridge the ligand-RAGE axis upregulation with increased oxidant stress and inflammation.

CoQ10 is known as a powerful endogenous lipophilic antioxidant [59] that directly protects cellular components from free radicals and indirectly regenerates other antioxidants [60]. This fact explains the restoration of GSH and the decrease of lipid peroxidation proved by the current model and others [57], [58], in addition to the inhibition of MPO and the elevation of sRAGE. In a vicious cycle, the CoQ10-mediated sRAGE elevation adds to its antioxidant mechanisms, where inhibition of AGE/RAGE axis entails an elevation in sRAGE and a decrease in the production of ROS [55]. Additionally, the CoQ10 inhibitory action on MPO can be another reason for decreasing AGEs production as mentioned previously [56]. Of note, inhibition of neutrophils recruitment by CoQ10 along with ROS can favor the improved insulin sensitivity. Our results coincide with those of Kunitomo et al. [61] and Tsai et al. [62], who documented that CoQ10 significantly attenuated the increase of oxidative and nitrative stress markers, oxidized LDL, MPO and mitigated the NF-κB and downstream inflammatory mediators, including the expression of adhesion molecules. Inhibition of the latter molecules can further explain the CoQ10-induced low MPO activity.

Moreover, diabetic animals showed hypo-adiponectinemia that may result from obesity-induced IR in adipose tissue, especially the visceral ones [63], which initiates further metabolic alterations in other peripheral tissues, viz., liver and skeletal muscles [64]. Moreover, Krssak et al. [65] and Shulman [66] stated that the elevated plasma FFAs and TGs in muscles lead to the development of muscle IR, events that correlate, even partially, to hypoadiponectenimea. Not knowing if altered adiponectin is a cause or a consequent, hypoadiponectinemia was reported to down regulate adiponectin receptors in the skeletal muscles of ob/ob mice [67], and livers of IR rats [19], findings that accentuate the present decrease in the adiponectin receptors in both organs. On the other hand, other studies [67], [68] concluded that the decreased levels of adiponectin receptors participate in the development of hypoadiponectinemia with the consequent IR. Additionally, hypoadiponectinemia may be responsible for the pan dyslipidemia observed in this work, where high adiponectin level enhances fatty acids oxidation, reduces muscular TGs, improves muscle fat burn, insulin sensitivity and lowers hepatic glucose output, effects that are abated by hypoadiponectenemia. Therefore, the ability of CoQ10 and glimepiride to increase the levels of adiponectin and its receptors, in this study, verify another mechanism for their insulin sensitizing effect along with their combination, which showed a better significant effect. The effect of CoQ10 overrides that of glimepiride in correcting the disturbed lipid profile, a finding that may be linked to its action on the adiponectin level.

Another adipocytokine assessed in the current study was the pro-inflammatory visfatin, which was elevated significantly in the present model and previous studies [69], reporting its elevation in many inflammatory diseases including obesity, IR and type 2 diabetes [70]. Visfatin was previously thought to increase in diabetic patients to enhance glucose uptake *in vitro* and *in vivo via* its binding to insulin receptor, causing its phosphorylation, as well as the IRS-1 and -2 [71]. However, to share the ambiguity of the other adipocytokines in their metabolic and immune functions, visfatin was found to bind to and activate the insulin receptor, but insulin does not interact with its cytokine-inducing effects [72]. Moreover, it was proven that visfatin plays a pivotal role in the generation of IR, by inducing the expression and production of inflammatory mediators, *viz.*, IL-1β, TNF-α, and IL-6 *via* NF-κB activation [70], [72]. Additionally, increased visfatin level offers another explanation for the increased neutrophils infiltration, where administration of visfatin inhibitor succeeded to decrease MPO level [73]. Moreover, visfatin was reported to assist leukocyte adhesion to endothelial cells by the induction of the cell adhesion molecules [70]. This effect may be mediated by the ROS-induced NF-κB activation; hence, linking oxidative stress with inflammatory pathways *via* the released adipocytokines [74]. Therefore, CoQ10 and glimepiride by inhibiting the level of visfatin highlight the improvement of insulin sensitivity, redox balance and inflammatory disorders. The effect of CoQ10 was better than that of glimepiride and the combination regimen offered a far better inhibitory effect on the visfatin content.

Perturbed lipid panel is known to elevate liver aminotransferases, a fact that is exemplified in the present study. Aminotransferases are indicators of hepatic health that is compromised in case of obesity, IR and diabetes [75]. Moreover, fatty changes are associated with the increased level of TNF-α, proven to increase in this model [19], which has a great influence on ALT elevation. Therefore, activated aminotransferases may reflect the role of hepatic gluconeogenesis and/or inflammation in the type 2 diabetes pathophysiology. Therefore, the inhibitory action of CoQ10 on ALT can reflect the inhibition in the hepatic gluconeogenesis and the type 2 associated inflammation indicating a better insulin sensitivity and correction of the diabetic disorders.

To this extent, the improving effect of CoQ10 on the glucose panel and insulin sensitivity can be attributed to its action on the insulin signaling pathway (insulin receptors, tyrosine kinase and PI3K) with the subsequent modulation in the function of the glucose transporters, as well as elevating sRAGE content and adiponectin with its receptors. Additionally, CoQ10 extended its positive effect by inhibiting ROS, MPO and visfatin. The study, hence, highlighted for the first time the possible mechanisms responsible for the CoQ10-mediated insulin sensitivity and its antidiabetic action. These findings support its useful effect as an add-on supplement with known antidiabetic drugs.

## References

[1] Keller KB, Lemberg L (2003) Obesity and the metabolic syndrome. Am J Crit Care. 12: 167–170. 12625176

[2] Schaalan M, El-Abhar H, Barakat M, El-Denshary ES (2009) Westernized-like-diet-fed rats: effect on glucose homeostasis, lipid profile, and adipocyte hormones and their modulation by rosiglitazone and glimepiride. J Diabetes Complications 23: 199–208. 18407527 10.1016/j.jdiacomp.2008.02.003

[3] Lamson DW, Plaza SM (2002) Mitochondrial factors in the pathogenesis of diabetes: a hypothesis for treatment. Altern Med Rev 7: 94–111. 11991790

[4] Van de Weijer T, Sparks LM, Phielix E, Meex RC, van Herpen NA, et al (2013) Relationships between mitochondrial function and metabolic flexibility in type 2 diabetes mellitus. PLoS One 8: e51648. 23418416 10.1371/journal.pone.0051648 PMC3572106

[5] Lee HM, Kim JJ, Kim HJ, Shong M, Ku BJ, et al (2013) Up-regulated NLRP3 inflammasome activation in patients with Type 2 Diabetes. Diabetes 62: 194–204. 23086037 10.2337/db12-0420 PMC3526026

[6] Sourris KC, Harcourt BE, Tang PH, Morley AL, Huynh K, et al (2012) Ubiquinone (coenzyme Q10) prevents renal mitochondrial dysfunction in an experimental model of type 2 diabetes. Free Radic Biol Med 52: 716–723. 22172526 10.1016/j.freeradbiomed.2011.11.017

[7] Eriksson J, Lindström J, Valle T, Aunola S, Hämäläinen H, et al (1999) Prevention of type II diabetes in subjects with impaired glucose tolerance: the Diabetes Prevention Study (DPS) in Finland: Study design and 1-year interim report on the feasibility of the lifestyle intervention programme. Diabetologia 42: 793–801. 10440120 10.1007/s001250051229

[8] Sena CM, Nunes E, Gomes A, Santos MS, Proença T, et al (2008) Supplementation of coenzyme Q10 and alpha-tocopherol lowers glycated hemoglobin level and lipid peroxidation in pancreas of diabetic rats. Nutr Res 28: 113–121. 19083397 10.1016/j.nutres.2007.12.005

[9] Villalba JM, Parrado C, Santos-Gonzalez M, Alcain FJ (2010) Therapeutic use of coenzyme Q10 and coenzyme Q10-related compounds and formulations. Expert Opin Investig Drugs 19: 535–554. 10.1517/13543781003727495 20367194

[10] Molyneux SL, Florkowski CM, George PM, Pilbrow AP, Frampton CM, et al (2008) Coenzyme Q10: an independent predictor of mortality in chronic heart failure. J Am Coll Cardiol 52: 1435–1441. 19017509 10.1016/j.jacc.2008.07.044

[11] Chew GT, Watts GF (2004) Coenzyme Q10 and diabetic endotheliopathy: oxidative stress and the ‘recoupling hypothesis’. QJM. 97: 537–548. 10.1093/qjmed/hch089 15256611

[12] Sohet FM, Neyrinck AM, Pachikian BD, de Backer FC, Bindels LB, et al (2009) Coenzyme Q10 supplementation lowers hepatic oxidative stress and inflammation associated with diet-induced obesity in mice. Biochem Pharmacol 78: 1391–1400. 19632207 10.1016/j.bcp.2009.07.008

[13] Ahmadvand H, Tavafi M, Khosrowbeygi A (2012) Amelioration of altered antioxidant enzymes activity and glomerulosclerosis by coenzyme Q10 in alloxan-induced diabetic rats. J Diabetes Complications 26: 476–482. 22795334 10.1016/j.jdiacomp.2012.06.004

[14] Andersen CB, Henriksen JE, Hother-Nielsen O, Vaag A, Mortensen SA, et al (1997) The effect of coenzyme Q10 on blood glucose and insulin requirement in patients with insulin dependent diabetes mellitus. Mol Aspects Med 18 Suppl: S307–309 10.1016/s0098-2997(97)00010-1 9266541

[15] Hodgson JM, Watts GF, Playford DA, Burke V, Croft KD (2002) Coenzyme Q10 improves blood pressure and glycaemic control: a controlled trial in subjects with type 2 diabetes. Eur J Clin Nutr 56: 1137–1142. 12428181 10.1038/sj.ejcn.1601464

[16] Mezawa M, Takemoto M, Onishi S, Ishibashi R, Ishikawa T, et al (2012) The reduced form of coenzyme Q10 improves glycemic control in patients with type 2 diabetes: an open label pilot study. Biofactors 38: 416–421. 22887051 10.1002/biof.1038

[17] Bauerova K, Paulovicova E, Mihalova D, Drafi F, Strosova M, et al (2010) Combined methotrexate and coenzyme Q10 therapy in adjuvant-induced arthritis evaluated using parameters of inflammation and oxidative stress. Acta Biochimica Polonica 57: 347–354. 20827446

[18] Matthews DR, Hosker JP, Rudenski AS, Naylor BA, Treacher DF, et al (1985) Homeostasis model assessment: Insulin resistance and b-cell function from fasting plasma glucose and insulin concentrations in man. Diabetologia 28: 412–419. 3899825 10.1007/BF00280883

[19] El-Abhar HS, Schaalan MF (2012) Topiramate-induced modulation of hepatic molecular mechanisms: An aspect for its anti-insulin resistant effect. PLoS ONE 7: e37757 doi:10.1371/journal.pone.0037757 22649556 10.1371/journal.pone.0037757 PMC3359316

[20] Bradford MM (1976) A rapid and sensitive method for the quantitation of microgram quantities of protein utilizing the principle of protein-dye binding. Anal Biochem 72: 248–254. 942051 10.1016/0003-2697(76)90527-3

[21] Corin RE, Donner DB (1982) Insulin receptors convert to a higher affinity state subsequent to hormone binding. A two state model for the insulin receptors. J Biol Chem 275: 104–110. 7031059

[22] Miller JL, Lynn CH, Shuster J, Driscoll DJ (2011) Carnitine and coenzyme Q10 levels in individuals with Prader-Willi syndrome. Am J Med Genet A 155 A: 569–573. 10.1002/ajmg.a.33887 PMC3285456 21337696

[23] Dzugkoev SG, Kaloeva MB, Dzugkoeva FS (2012) Effect of combination therapy with coenzyme Q10 on functional and metabolic parameters in patients with type 1 diabetes mellitus. Bull Exp Biol Med 152: 364–366.78. 22803087 10.1007/s10517-012-1529-7

[24] Mc Carty MF (1999) Can correction of sub-optimal coenzyme Q status improve beta-cell function in type II diabetics? Med Hypotheses 52: 397–400. 10416946 10.1054/mehy.1997.0681

[25] Crescenzo R, Bianco F, Falcone I, Tsalouhidou S, Yepuri G, et al (2012) Hepatic mitochondrialenergetics during catch-up fat with high-fat diets rich in lard or safflower oil. Obesity (SilverSpring) 20: 1763–1772. 10.1038/oby.2011.167 21720434

[26] Ernster L, Dallner G (1995) Biochemical, physiological and medical aspects of ubiquinone function. Biochim Biophys Acta 1271: 195–204. 7599208 10.1016/0925-4439(95)00028-3

[27] Moore MC, Cherrington AD, Mann SL, Davis SN (2000) Acute fructose administration decreases the glycemic response to an oral glucose tolerance test in normal adults. J Clin Endocrinol Metab 85: 4515–4519. 11134101 10.1210/jcem.85.12.7053

[28] Avramoglu RK, Basciano H, Adeli K (2006) Lipid and lipoprotein dysregulation in insulin resistant states. Clin Chim Acta 368: 1–19. 16480697 10.1016/j.cca.2005.12.026

[29] Julius U (2003) Influence of plasma free fatty acids on lipoprotein synthesis and diabetic dyslipidemia. Exp Clin Endocrinol Diabetes 111: 246–250. Review. 10.1055/s-2003-41284 12951628

[30] Taskinen MR, Beltz WF, Harper I, Fields RM, Schonfeld G, et al (1986) Effects of NIDDM on very-low-density lipoprotein triglyceride and apo-lipoprotein B metabolism. Studies before andafter sulfonylurea therapy. Diabetes 35: 1268–1277. 3530855 10.2337/diab.35.11.1268

[31] Song H, Shojima N, Sakoda H, Ogihara T, Fujishiro M, et al (2002) Resistin is regulated by C/EBPs, PPARs, and signal-transducing molecules. Biochem Biophys Res Commun 29: 291–298. 10.1016/s0006-291x(02)02551-2 12437985

[32] Marshall S, Bacote V, Traxinger RR (1991) Discovery of a metabolic pathway mediating glucose-induced desensitization of the glucose transport system. Role of hexosamine biosynthesis in the induction of insulin resistance. J Biol Chem 266: 4706–4712. 2002019

[33] McClain DA (2002) Hexosamines as mediators of nutrient sensing and regulation in diabetes. J Diabetes Complications 16: 72–80. 11872372 10.1016/s1056-8727(01)00188-x

[34] Ueno M, Bezerra RM, Silva MS, Tavares DQ, Carvalho CR, et al (2000) A high-fructose diet induces changes in pp185 phosphorylation in muscle and liver of rats. Braz J Med Biol Res 33: 1421–1427. 11105093 10.1590/s0100-879x2000001200004

[35] Mosthab L, Vogt B, Haring HU, Ullrich A (1991) Altered expression of insulin receptor types A and B in the skeletal muscle of noninsulin-dependent diabetes mellitus. Proc Natl Acad Sci USA 88: 4728–4730. 1711209 10.1073/pnas.88.11.4728 PMC51739

[36] Kellerer M, Sesti G, Seffer E, Obermaier-Kusser B, Pongratz DE, et al (1993) Altered pattern of insulin receptor isotypes in skeletal muscle membranes of typeII (noninsulin-dependent) diabetic subjects. Diabetologia 36: 628–632. 8359580 10.1007/BF00404072

[37] Sbraccia P, D’Adamo M, Leonetti F, Caiola S, Iozzo P, et al (1996) Chronic primary hyperinsulinemia is associated with altered insulin receptor mRNA splicing in muscle of patients with insulinoma. Diabetologia 39: 220–225. 8635675 10.1007/BF00403966

[38] Krauss H, Grzymisławski M, Koźlik J, Sosnowski P, Piatek J, et al (2004) The influence of glimepiride on the binding kinetics of insulin with its skeletal muscle and liver receptors in rats with short term and prolonged hyperglycemia induced by streptozotocin. Med Sci Monit 10: BR11–16. 14704628

[39] Baron AD, Zhu JS, Weldon H, Maianu L, Garvey WT (1995) Glucosamine induces insulin resistance in vivo by affecting GLUT-4 translocation in skeletal muscle: Implications for glucose toxicity. J Clin Invest 96: 2792–2801. 8675649 10.1172/JCI118349 PMC185989

[40] Stephens JM, Lee J, Pilch PF (1997) Tumor necrosis factor-alpha-induced insulin resistance in 3T3-L1 adipocytes is accompanied by a loss of insulin receptor substrate-1 and GLUT4 expression without a loss of insulin receptor-mediated signal transduction. J Biol Chem 272: 971–976. 8995390 10.1074/jbc.272.2.971

[41] Leguisamo NM, Lehnen AM, Machado UF, Okamoto MM, Markoski MM, et al (2012) GLUT4 content decreases along with insulin resistance and high levels of inflammatory markers in rats with metabolic syndrome. Cardiovasc Diabetol 11: 100. 22897936 10.1186/1475-2840-11-100 PMC3439702

[42] Bernat-Karpińska M, Piątkiewicz P, Czech A, Wierzbicki P (2012) The expression of particular glucose transporters and insulin resistance indicators in the risk groups of type 2 diabetes–a two year follow-up. Endokrynol Pol 63: 212–219. [Abstract]. 22744628

[43] Ho YJ, Chen WP, Chi TC, Chang Chien CC, Lee AS, et al (2013) 1 Caffeic acid phenethyl amide improves glucose homeostasis and attenuates the progression of vascular dysfunction in Streptozotocin-induced diabetic rats. Cardiovasc Diabetol 12: 99. 23829275 10.1186/1475-2840-12-99 PMC3706244

[44] Kruszynska YT, Worrall DS, Ofrecio J, Frias JP, Macaraeg G, et al (2002) Fatty acid-induced insulin resistance: decreased muscle PI3K activation but unchanged Akt phosphorylation. J Clin Endocrinol Metab 87: 226–234. 11788651 10.1210/jcem.87.1.8187

[45] Choi H, Park HH, Lee KY, Choi NY, Yu HJ, et al (2013) Coenzyme Q10 restores amyloid beta-inhibited proliferation of neural stem cells by activating the PI3K pathway. Stem Cells Dev. 22: 2112–2120. 10.1089/scd.2012.0604 23509892

[46] Ma P, Xiong W, Liu H, Ma J, Gu B, et al (2011) Extrapancreatic roles of glimepiride on osteoblasts from rat manibular bone in vitro: Regulation of cytodifferentiation through PI3-kinases/Akt signalling pathway. Arch Oral Biol. 56: 307–316. 10.1016/j.archoralbio.2010.10.009 21055727

[47] Oka Y, Asano T, Shibasaki Y, Lin JL, Tsukuda K, et al (1990) Increased liver glucose transporter protein and mRNA in streptozocin-induced diabetic rats. Diabetes 39: 441–446. 2180755 10.2337/diab.39.4.441

[48] Burcelin R, Eddouks M, Kande J, Assan R, Girard J (1992) Evidence that GLUT-2 mRNA and protein concentrations are decreased by hyperinsulinaemia and increased by hyperglycaemia in liver of diabetic rats. Biochem J 288: 675–679. 1463468 10.1042/bj2880675 PMC1132064

[49] Postic C, Burcelin R, Rencurel F, Pegorier JP, Loizeau M, et al (1993) Evidence for a transient inhibitory effect of insulin on GLUT2 expression in the liver: studies in vivo and in vitro. Biochem J 293: 119–124. 8328952 10.1042/bj2930119 PMC1134328

[50] Bernsmeier C, Heim MH (2009) Insulin resistance in chronic hepatitis C: mechanisms and clinical relevance. Swiss Med Wkly 139: 678–84. 20047129 10.4414/smw.2009.12765

[51] Koyama H, Yamamoto H, Nishizawa Y (2007) Endogenous secretory RAGE as a novel biomarker for metabolic syndrome and cardiovascular diseases. Biomark Insights 2: 331–339. 19662215 PMC2717812

[52] Tan KC, Shiu SW, Wong Y, Tam X (2011) Serum advanced glycation end products (AGEs) are associated with insulin resistance. Diabetes Metab Res Rev 27: 488–492. 21337488 10.1002/dmrr.1188

[53] Lu L, Peng W, Wang W, Wang L, Chen Q, et al (2011) Effects of atorvastatin on progression of diabetic nephropathy and local RAGE and soluble RAGE expressions in rats. J Zhejiang Univ Sci B 2: 652–659. 10.1631/jzus.B1101004 PMC3150719 21796806

[54] Koyama H, Shoji T, Yokoyama H, Motoyama K, Mori K, et al (2005) Plasma level of endogenous secretory RAGE is associated with components of the metabolic syndrome and atherosclerosis. Arterioscler Thromb Vasc Biol 25: 2587–2593. 16224056 10.1161/01.ATV.0000190660.32863.cd

[55] Devangelio E, Santilli F, Formoso G, Ferroni P, Bucciarelli L, et al (2007) Soluble RAGE in type 2 diabetes: association with oxidative stress. Free Radic Biol Med 43: 511–518. 17640561 10.1016/j.freeradbiomed.2007.03.015

[56] Anderson JW, Gowri MS, Turner J, Nichols L, Diwadkar VA, et al (1999) Antioxidant supplementation effects on low-density lipoprotein oxidation for individuals with type 2 diabetes mellitus. J Am Coll Nutr 18: 451–461. 10511327 10.1080/07315724.1999.10718883

[57] El-ghoroury EA, Raslan HM, Badawy EA, El-Saaid GS, Agybi MH, et al (2009) Malondialdehyde and coenzyme Q10 in platelets and serum in type 2 diabetes mellitus: correlation with glycemic control. Blood Coagul Fibrinolysis 20: 248–251. 19530339 10.1097/mbc.0b013e3283254549

[58] Dzugkoev SG, Kaloeva MB, Dzugkoeva FS (2012) Effect of combination therapy with coenzyme Q10 on functional and metabolic parameters in patients with type 1 diabetes mellitus. Bull Exp Biol Med 152: 364–366. 22803087 10.1007/s10517-012-1529-7

[59] Bhagavan HN, Chopra RK (2007) Plasma coenzyme Q10 response to oral ingestion of coenzyme Q10 formulations. Mitochondrion 7: S78–88. 17482886 10.1016/j.mito.2007.03.003

[60] Crane FL (2001) Biochemical functions of coenzyme Q10. J Am Coll Nutr 20: 591–598. 11771674 10.1080/07315724.2001.10719063

[61] Kunitomo M, Yamaguchi Y, Kagota S, Otsubo K (2008) Beneficial effect of coenzyme Q10 on increased oxidative and nitrative stress and inflammation and individual metabolic components developing in a rat model of metabolic syndrome. J Pharmacol Sci 107: 128–137. 18544898 10.1254/jphs.fp0072365

[62] Tsai KL, Chen LH, Chiou SH, Chiou GY, Chen YC, et al (2012) Coenzyme Q10 suppresses oxLDL-induced endothelial oxidative injuries by the modulation of LOX-1-mediated ROS generation via the AMPK/PKC/NADPH oxidase signaling pathway. Mol Nutr Food Res 55: S227–240. 10.1002/mnfr.201100147 21812107

[63] Milan G, Granzotto M, Scarda A, Calcagno A, Pagano C, et al (2002) Resistin and adiponectin expression in visceral fat of obese rats: effect of weight loss. Obes Res 10: 1095–1103. 12429872 10.1038/oby.2002.149

[64] Lu JY, Huang KC, Chang LC, Huang YS, Chi YC, et al (2008) Adiponectin: a biomarker of obesity-induced insulin resistance in adipose tissue and beyond. J Biomed Sci 15: 565–576. 18535923 10.1007/s11373-008-9261-z

[65] Krssak M, Falk Petersen K, Dresner A, Di Pietro L, Vogel SM, et al (1999) Intramyocellular lipid concentrations are correlated with insulin sensitivity in humans: a 1H NMR spectroscopy study. Diabetologia 42: 113–116. 10027589 10.1007/s001250051123

[66] Shulman GI (2000) Cellular mechanisms of insulin resistance. J Clin Invest 106: 171–176. 10903330 10.1172/JCI10583 PMC314317

[67] Kadowaki T, Yamauchi T (2005) Adiponectin and Adiponectin Receptors. Endocrine Reviews 26: 439–451. 15897298 10.1210/er.2005-0005

[68] Yamauchi T, Nio Y, Maki T, Kobayashi M, Takazawa T, et al (2007) Targeted disruption of AdipoR1 and AdipoR2 causes abrogation of adiponectin binding and metabolic actions. Nat Med 13: 332–339. 17268472 10.1038/nm1557

[69] Haider DG, Schaller G, Kapiotis S, Maier C, Luger A, et al (2006) The release of the adipocytokine visfatin is regulated by glucose and insulin. Diabetologia 49: 1909–1914. 16736128 10.1007/s00125-006-0303-7

[70] Lee WJ, Wu CS, Lin H, Lee IT, Wu CM, et al.. (2009) Visfatin-induced expression of inflammatory mediators in human endothelial cells through the NF-κB pathway. Int J Obes 33: 30 465–472. 10.1038/ijo.2009.24 19223849

[71] Fukuhara A, Matsuda M, Nishizawa M, Segawa K, Tanaka M, et al (2005) Visfatin: a protein secreted by visceral fat that mimics the effects of insulin. Science 307: 426–430. 15604363 10.1126/science.1097243

[72] Moschen AR, Kaser A, Enrich B, Mosheimer B, Theurl M, et al (2007) Visfatin, an adipocytokine with proinflammatory and immunomodulating properties. J Immunol 178: 1748–1758. 17237424 10.4049/jimmunol.178.3.1748

[73] Esposito E, Impellizzeri D, Mazzon E, Fakhfouri G, Rahimian R, et al (2012) The NAMPT inhibitor FK866 reverts the damage in spinal cord injury. J Neuroinflammation 9: 66. 22490786 10.1186/1742-2094-9-66 PMC3353188

[74] Kim SR, Bae YH, Bae SK, Choi KS, Yoon KH, et al (2008) Visfatin enhances ICAM-1 and VCAM-1 expression through ROS-dependent NF-kappaB activation in endothelial cells. Biochim Biophys Acta 1783: 886–895. 18241674 10.1016/j.bbamcr.2008.01.004

[75] Vozarova B, Stefan N, Lindsay RS, Saremi A, Pratley RE, et al (2002) High alanine aminotransferase is associated with decreased hepatic insulin sensitivity and predicts the development of type 2 diabetes. Diabetes 51: 1889–1895. 12031978 10.2337/diabetes.51.6.1889

